# H3K27 tri-demethylase JMJD3 inhibits macrophage apoptosis by promoting ADORA2A in lipopolysaccharide-induced acute lung injury

**DOI:** 10.1038/s41420-022-01268-y

**Published:** 2022-12-01

**Authors:** Yizhuo Gao, Na Wang, Dong Jia

**Affiliations:** 1 Department of Pulmonary and Critical Care Medicine, Shengjing Hospital of China Medical University, No. 36, Sanhao Street, Shenyang, China; 2 Occupational Disease and Occupational Health Prevention and Control Institute, Liaoning Centre for Disease Control and Prevention, Shenyang, Liaoning China; 3 Department of Emergency Medicine, Shengjing Hospital of China Medical University, No. 36, Sanhao Street, Shenyang, China

**Keywords:** Apoptosis, Acute inflammation

## Abstract

Acute lung injury (ALI) is a common critical disease, which is characterized by an uncontrolled, acute inflammatory response, diffuse lung damage and ultimately directly deteriorates into acute respiratory distress syndrome. The number of pro-inflammatory macrophages is related to the severity of ALI. Up-regulation of lipopolysaccharide (LPS)-activated macrophage apoptosis can reduce the pro-inflammatory reactions. Jumonji domain-containing protein D3 (JMJD3)-mediated histone 3 lysine 27 trimethylation (H3K27me3) demethylation may promote the pro-inflammatory response of macrophages under LPS stimulation. However, the mechanism of JMJD3 affecting macrophage apoptosis is still not clear. To explore this gap in knowledge, the ALI mice model with intratracheal administration of LPS and RAW264.7 cells with LPS stimulation were used as in vivo and in vitro experiments. The expression of JMJD3 and H3K27me3 and their cellular localization were analysed in lung tissue. Apoptosis was evaluated using TUNEL staining and flow cytometry. Expression of H3K27me3, ADORA2A and C/EBPβ were compared among different treatments and chromatin immunoprecipitation was performed to investigate the regulatory relationship. Our study showed that JMJD3 expression was upregulated in LPS-induced ALI mice and RAW264.7 cells. JMJD3-indued H3K27me3 demethylation inhibited caspase-3 cleavage by upregulating ADORA2A to decrease LPS-stimulated macrophage apoptosis and promoted the inflammatory reaction. This H3K27me3 demethylation also increased C/EBPβ expression, which may enhance ADORA2A expression further. Besides, inhibiting ADORA2A can also promote LPS-limited macrophage apoptosis. Moreover, the inhibition of JMJD3 in vivo and in vitro relieved the inhibition of macrophage apoptosis thus leading to the resolution of the inflammation. JMJD3 might inhibit macrophage apoptosis by promoting ADORA2A expression in LPS-induced ALI.

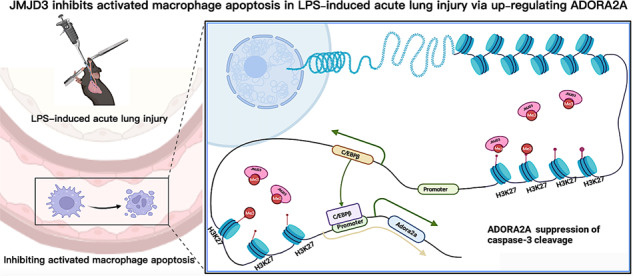

## Introduction

Acute lung injury (ALI) is a critical entity of acute respiratory failure in clinical that is associated with substantial morbidity and mortality in critically ill patients [1]. Therefore, exploring the underlying pathophysiological mechanisms of ALI would aid in the development of effective treatments for this condition.

Macrophages play multiple roles in ALI. Lipopolysaccharide (LPS)-stimulated macrophages to promote inflammation and ALI development. The apoptosis of activated macrophages is inhibited in the response to LPS stimulation [2] which aggravates the inflammatory reaction [3]. Conversely, the enhancement of LPS-stimulated macrophages’ apoptosis relieves acute inflammation [3, 4]. When ALI is triggered, the epithelial–endothelial barrier is disrupted [5]. This facilitates macrophage influx into the alveolar space, which is enhanced by LPS, thus aggravating inflammation and ALI. These influent macrophages lead to increased numbers of macrophages in the lung under LPS stimulation. This positive feedback amplifies inflammation [5]. Hence, terminating this positive feedback of macrophages on inflammation by promoting macrophages’ apoptosis could provide novel potentials in ALI treatment. Based on the pathophysiology described above, we hypothesize that promoting the apoptosis of activated macrophages might assist in ALI treatment.

Jumonji domain-containing protein D3 (JMJD3) is a specific demethylase of histone 3 lysine 27 trimethylation (H3K27me3) [6] and was promoted by LPS in macrophage [7]. H3K27me3 is an epigenetic modification of histones that alters the three-dimensional conformation of chromatin and this methylation/demethylation has been shown to be involved in the inflammation produced by macrophages [8, 9]. JMJD3 induces the demethylation of H3K27me3 and regulates multiple pathophysiological processes in macrophages [7]. However, the role of JMJD3-induced demethylation in the regulation of macrophage apoptosis remains to be elucidated. Additionally, the role of JMJD3 in activated macrophage apoptosis following LPS-induced ALI is poorly understood.

ADORA2A suppresses macrophage apoptosis by inhibiting the expression of tumour necrosis factor-α (TNF-α) [10, 11], an inflammatory cytokine that is released by activated macrophages [12]. However, the role of *Adora2a* in ALI is heavily debated [10, 13, 14]. C/EBPβ is a transcription factor that regulates inflammation caused by JMJD3-induced H3K27me3 demethylation [15] and shows a correlation with apoptosis in macrophages [16]. C/EBPβ and ADORA2A might both be regulated by JMJD3, but their role in ALI-induced ALI and its regulation relationship is not fully understood.

Our study aims to explore the effect of JMJD3 on ADORA2A expression and its regulatory role in macrophage apoptosis when ALI provides a new mechanism for JMJD3 in ALI and is a potential target for anti-inflammation therapy.

## Results

### JMJD3 expression was upregulated in lung tissue macrophages of ALI

**Fig. 1:**
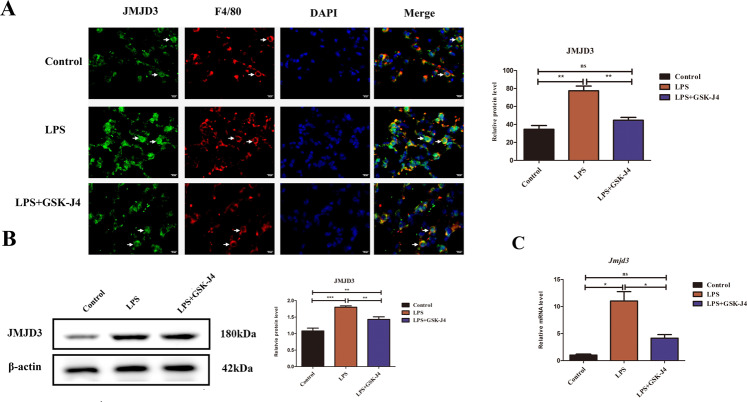
JMJD3 was upregulated in lung tissue of ALI and LPS-stimulated macrophage. **A** JMJD3 expression level in the LPS group was higher than that in the control group but lower than that in the GSK-J4 treatment group (*n* = 10 in each group*, P* < 0.01, control vs. LPS group, *P* < 0.01, LPS vs. LPS+GSK-J4 group). **B** Western blot shows that in macrophage, the JMJD3 expression level in the LPS group was higher than that in the control group (*P* < 0.01, control vs. LPS group), using GSK-J4 could inhibit JMJD3 in macrophage (*n* = 10 in each group*, P* < 0.01, LPS vs. LPS+GSK-J4 group). **C** Transcription level of *Jmjd3* in the LPS group was higher than that in the control group (*P* < 0.01, control vs. LPS group) but lower than that in GSK-J4 treatment group (*P* < 0.01, LPS vs. LPS + GSK-J4 group). ns indicated no statistical significance, *P < 0.05, **P < 0.01, and ***P < 0.001.

An experimental animal model of ALI was performed in the LPS choking on the trachea of C57BL/6j mice, and GSK-J4(JMJD3 specific inhibitor) was used as an intervention to explore the effect of inhibition of JMJD3 on ALI. In lung tissue macrophages, JMJD3 expression in LPS-induced ALI was higher than that in the control group, and using GSK-J4 to treat LPS-induced ALI can attenuate the JMJD3 expression (Fig. 1A). In LPS-stimulated macrophage, JMJD3 protein and mRNA in LPS group were higher than those in the control group and were lower than those in GSK-J4 group (Fig. 1B, C). LPS promoted JMJD3 expression in vivo and in vitro.

### Inhibiting JMJD3 relieved lung tissue’s pathological performance and LPS-induced inflammation

**Fig. 2:**
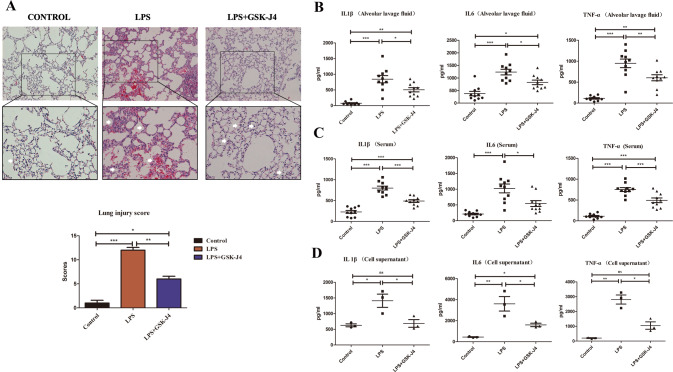
Using GSK-J4 to inhibit JMJD3 could relieve ALI-related pathological change and inflammation. H&E staining of lung tissues mice (*n* = 10 in each group) which were administered 2.5 mg/kg LPS intratracheally developed more alveolar collapse and inflammatory cell aggregation in histologic findings consistent with acute lung injury pathological changes. Treatment with either GSK-J4 alleviated these lung injury-associated inflammatory effects in the LPS-treated mice. The lung injury histopathologic score in the LPS group was higher than that in the control group. It was significantly lower in the group where LPS was added with either GSK-J4 compared to LPS alone (*P* < 0.001, control vs. LPS group, *P* < 0.01, LPS vs. LPS+GSK-J4 group). **A** IL-1β, IL-6, and TNF-α in blood sera (*n* = 10 in each group). **B** IL-1β, IL-6, and TNF-α in alveolar lavage fluids (*n* = 10 in each group). **C** IL-1β, IL-6, and TNF-α in serum (*n* = 10 in each group). **D** IL-1β, IL-6, and TNF-α in cell supernatants (*n* = 3 in each group). ns indicated no statistical significance, *P < 0.05, **P < 0.01, and ***P < 0.001.

The pathological changes in mice lung tissue including pulmonary consolidation, alveolar collapse, and inflammatory cell infiltration were used to the change of ALI. The lungs of mice exposed to intratracheal LPS showed an exacerbation of ALI. When treatment with GSK-J4, these pathological changes were relieved, as observed by H&E staining results. (Fig. 2A). The histopathological score in the LPS group was higher than that in the control group and GSK-J4 treatment makes the score reduced. LPS can lead to serious lung injury and inhibiting JMJD3 can relieve the ALI.

To evaluate the degree of the inflammatory response to ALI, we detected the levels of inflammatory cytokine expression in bronchoalveolar lavage fluid and serum, including the detection of IL1β, IL6, and TNF-α. The inflammatory cytokines of IL1β, IL6, and TNF-α were measured by ELISA in BAL fluid and serum in vivo. In the LPS group, the expression levels of inflammatory cytokines were higher than those in the control group in vivo (Fig. 2B, C). Using GSK-J4 treatment reduced the level of inflammatory cytokines (Fig. 2B, C). A in vitro experiment was performed to simulate the in vivo experiments using LPS stimulating in RAW264.7 cell. The results revealed in the LPS group, the expression levels of inflammatory cytokines were higher than those in the control group in vivo (Fig. 2D). In GSK-J4 treatment group, the level of inflammatory cytokines was lower than that in the LPS group (Fig. 2D). LPS promoted the ALI mice and LPS-stimulated macrophage’s inflammation and GSK-J4 treatment can decrease the inflammatory reaction.

### JMJD3-induced H3K27me3 demethylation and up-regulated ADORA2A and C/EBPβ expression

**Fig. 3:**
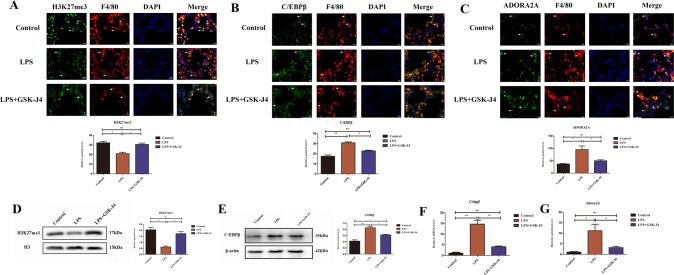
JMJD3-induced H3K27me3 demethylation and up-regulated ADORA2A and C/EBPβ expressions. **A** H3K27me3 expression in the LPS group was lower than that in the control group (*P* < 0.05, control vs. LPS group), and inhibiting JMJD3 with GSK-J4 showed higher H3K27me3 expression (*P* < 0.05, LPS vs. LPS+GSK-J4 group) (*n* = 10 in each group). **B** C/EBPβ expression in the LPS group was higher than that in the control group(*P* < 0.01, control vs. LPS group,), inhibiting JMJD3 downregulated C/EBPβ expression (*P* < 0.05, LPS vs. LPS+GSK-J4 group) (*n* = 10 in each group). **C** ADORA2A expression in the LPS group was higher than that in the control group (*P* < 0.05, control vs. LPS group), inhibiting JMJD3 downregulated ADORA2A expression (*P* < 0.05, LPS vs. LPS+GSK-J4 group) (*n* = 10 in each group). **D** Western blot shows that the H3K27me3 expression level in the LPS group was lower than that in both the control group and GSK-J4 treatment group (*P* < 0.05, control vs. LPS group, *P* < 0.05, LPS vs. LPS+GSK-J4 group). **E** C/EBPβ expression level in the LPS group was higher than that in the control group and inhibiting JMJD3 make C/EBPβ lower. (*P* < 0.01, control vs. LPS group, *P* < 0.05, LPS vs. LPS + GSK-J4 group). **F** QPCR shows that the transcription level of *C/ebpβ* in the LPS group was higher than that in the control group using the GSK-J4 treatment group with lower *C/ebpβ* (*P* < 0.01, control vs. LPS group, *P* < 0.01, LPS vs. LPS+GSK-J4 group). **G** QPCR shows that the transcription level of *Adora2a* in the LPS group was higher than that in the control group using the GSK-J4 treatment group with lower *Adora2a.* ns indicated no statistical significance, *P < 0.05, **P < 0.01, and ***P < 0.001.

Considering the specific regulatory relationships in molecular structure, in order to test whether H3K27me3 was involved in the JMJD3 pathway when ALI. After locating macrophages, the results of in vivo immunofluorescence analysis revealed that in the LPS group, H3K27me3 expression was lower than that in the control group and the expression of H3K27me3 was down-regulated by GSK-J4-inhibiting JMJD3 (Fig. 3A). Besides, the expression of ADORA2A and C/EBPβ were higher than those in the control group and using GSK-J4 could reduce their expression than those in LPS group (Fig. 3B, C). In vitro, western blot revealed that H3K27me3 expression in the LPS group was lower than that in the control group and the GSK-J4 treatment group was higher than that in the LPS group (Fig. 3D). However, ADORA2A and C/EBPβ protein and mRNA were higher than those in the control group. GSK-J4 treatment down-regulates the expression of ADORA2A and C/EBPβ (Fig. 3E–G). Overall, in vivo and in vitro, JMJD3 inhibited macrophage’s H3K27me3 and upregulated the ADORA2A and C/EBPβ expressions in macrophage of ALI lung and LPS-stimulated RAW264.7 cells.

**Fig. 4:**
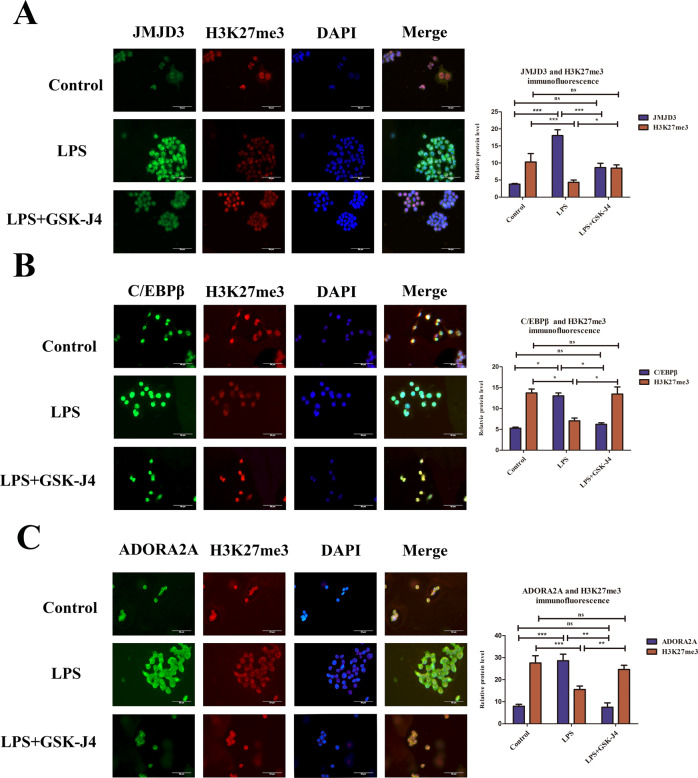
JMJD3-induced H3K27me3 demethylation and up-regulated ADORA2A and C/EBPβ expressions in macrophage. **A** In LPS-stimulated macrophage, JMJD3 expression was increased while H3K27me3 was decreased (JMJD3: *P* < 0.001, control vs. LPS group; H3K27me3: *P* < 0.001, control vs. LPS group),using GSK-J4 inhibit JMJD3 with lower JMJD3 and higher H3K27me3 (JMJD3: *P* < 0.05, LPS vs. LPS + GSK-J4 group;H3K27me3: *P* < 0.001, LPS vs. LPS + GSK-J4 group) (*n* = 3 in each group). **B** In the LPS group, C/EBPβ expression was increased, and inhibiting JMJD3 could reduce C/EBPβ expression (C/EBPβ: *P* < 0.05, control vs. LPS group, *P* < 0.05, LPS vs. LPS+GSK-J4 group), (*n* = 3 in each group). **C** ADORA2A expression increased in LPS-stimulated macrophage and GSK-J4 made it downregulation (ADORA2A: *P* < 0.001, control vs. LPS group, *P* < 0.01, LPS vs. LPS + GSK-J4 group; H3K27me3: *P* < 0.001, control vs. LPS group, *P* < 0.01, LPS vs. LPS + GSK-J4 group) (*n* = 3 in each group). ns indicated no statistical significance, *P < 0.05, **P < 0.01, and ***P < 0.001.

To test the regulatory relationship and further explore the mechanism between JMJD3 and H3K27me3, H3K27me3 and C/EBPβ, H3K27me3 and ADORA2A protein expression, we conducted immunofluorescence analysis in macrophage. The results revealed that the expression of JMJD3, ADORA2A, and C/EBPβ proteins increased, whereas that of H3K27me3 decreased in the LPS group compared to the control group. Moreover, GSK-J4 inhibited JMJD3 and up-regulated H3K27me3, subsequently inhibiting ADORA2A, and C/EBPβthan those in the LPS group (Fig. 4A–C). JMJD3 as the H3K27me3 demethylase can reduce the H3K27me3 level. Besides, H3K27me3 as a sign of expression repression, expression decreased with C/EBPβ and ADORA2A expression increasing.

### The expression relationship between H3K27me3 and *C/ebpβ*, H3K27me3 and *Adora2a*

**Fig. 5:**
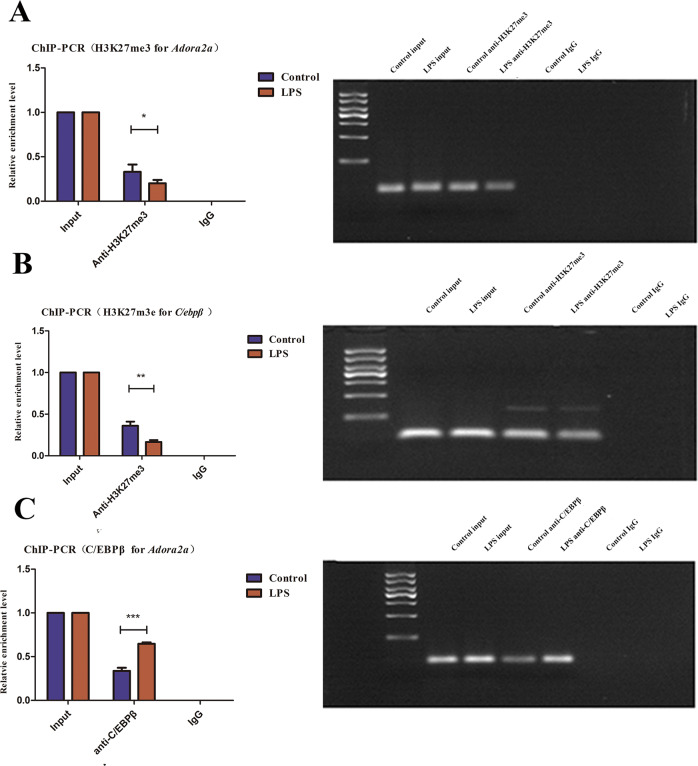
ChIP-PCR analysis for H3K27me3 and *C/ebpβ*, C/EBPβ and *Adora2a*. **A** The regulatory relationship between H3K27me3 and *Adora2a*: *Adora2a* relative enrichment level in the control group was higher than that in the LPS group (*n* = 3 in each group, *P* < 0.05, control vs. LPS group); **B** The regulatory relationship between H3K27me3 and *C/ebpβ*: *C/ebpβ* relative enrichment level in the control group was higher than that in LPS group (*n* = 3 in each group, *P* < 0.01, control vs. LPS group); **C** The regulatory relationship between C/EBPβ and *Adora2a*: *Adora2a* relative enrichment level in LPS group, was higher than that in the control group (*n* = 3 in each group, *P* < 0.001, control vs LPS group); ns indicated no statistical significance, *P < 0.05, **P < 0.01, and ***P < 0.001.

We found there were correlations between H3K27me3 and C/EBPβ, ADORA2A. Therefore, a ChIP-PCR was conducted to investigate these expression relationships, and the results have shown that H3K27me3 was enriched in *Adora2a* of the promoter region. In the control group, *Adora2a* relative enrichment level was approximately one time higher than that in the LPS group (Fig. 5A), while qPCR analysis revealed that *Adora2a* transcription in the LPS group was 10 times more than that in the control group. The expression relationship between H3K27me3 and *Adora2a* was not in accordance with qPCR results between the LPS and control groups. Therefore, we investigated the expression relationship between H3K27me3 and *C/ebpβ* expression. We found that H3K27me3 is enriched in *C/ebpβ* in the promoter region. In the control group, *C/ebpβ* relative enrichment level was approximately 1.5 times more than that in the LPS group (Fig. 5B). Moreover, C/EBPβ was enriched in the *Adora2a* promoter region in the LPS-treated group. In the LPS group, *Adora2a* relative enrichment level in LPS group was three times higher than that in the control group (Fig. 5C). In summary, LPS treatment may affect macrophage H3K27me3 levels and modulating the expression of *Adora2a* by directly promoting its transcription and amplifying *Adora2a* by promoting C/EBPβ. These findings were reflected in the qPCR analysis, where LPS treatment increased *Adora2a* expression in cells by approximately 10-fold compared to that in untreated cells.

### Inhibiting JMJD3 can promote macrophage apoptosis in lung tissue of ALI

**Fig. 6:**
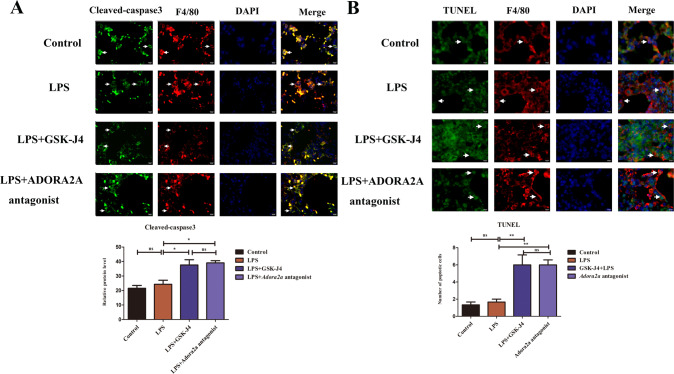
Inhibiting JMJD3 can promote macrophage apoptosis in lung tissue of ALI. **A** Cleaved caspase-3 expression in the LPS group was lower than those in GSK-J4 and ADORA2A antagonist groups (*n* = 10 in each group, *P* < 0.05, LPS vs LPS+GSK-J4 group, *P* < 0.05, LPS vs. LPS+*Adora2a* group). **B** The number of macrophage apoptosis in TUNEL of the LPS group was lower than those in GSK-J4 treatment and ADORA2A antagonist groups (*n* = 10 in each group, *P* < 0.01, LPS vs. LPS+GSK-J4 group, *P* < 0.01, LPS vs. LPS+*Adora2a* group). ns indicated no statistical significance, *P < 0.05, **P < 0.01, and ***P < 0.001.

After GSK-J4 treatment we found a decrease in the number of macrophages, then we further used cleaved-caspase3 and TUNEL to evaluate macrophage apoptosis in the ALI lung tissue. The results of immunofluorescence showed there were no statistical differences in the number of macrophage apoptosis in TUNEL and cleaved-caspase3 expression in control and LPS groups. These two above in the LPS group were lower than those in using GSK-J4 to inhibit JMJD3 and using specific antagonists to inhibit ADORA2A, which two can increase the macrophage’s apoptosis level (*P* < 0.05) (Fig. 6A, B).

### Inhibition of JMJD3 improved acute lung injury by down-regulating ADORA2A thereby promoting apoptosis of macrophages

**Fig. 7:**
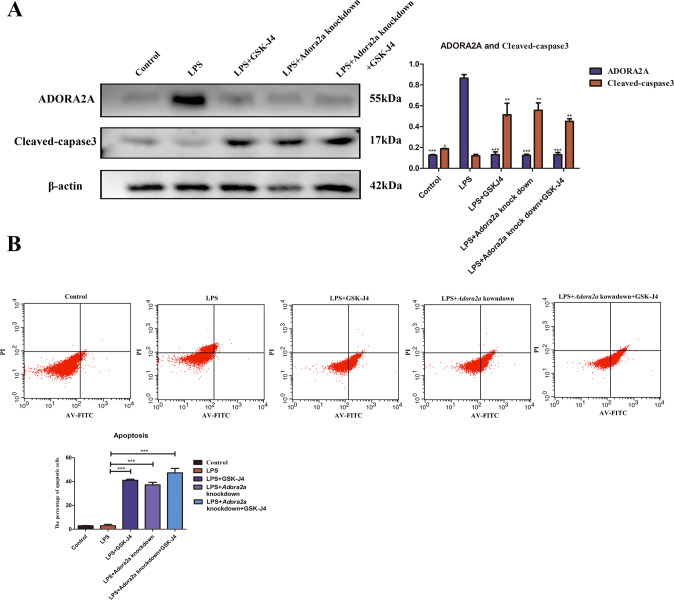
Inhibition of JMJD3 improved acute lung injury by down-regulating ADORA2A thereby promoting apoptosis of macrophages. **A** The relationship between ADORA2A and cleaved caspase-3 in western blots after *Adora2a* knockdown in LPS-stimulated RAW264.7 cells: ADORA2A expression in the LPS group was higher than that in both the control group and GSK-J4 group. Compared to cells that were only exposed to LPS, cells that were further treated with GSK-J4, underwent knockdown of *Adora2a*, or were both treated with GSK-J4 and underwent *Adora2a* knockdown showed higher levels of cleaved caspase 3 (*n* = 3 in each group, ADORA2A: *P* < 0.001, control vs. LPS group, *P* < 0.001, LPS+GSK-J4 vs. LPS group, *P* < 0.001, LPS+*Adora2a* knockdown vs. LPS group, *P* < 0.001, LPS+*Adora2a* knockdown+GSK-J4 vs LPS group. Cleaved caspase-3: *P* < 0.05, control vs. LPS group, *P* < 0.01, LPS+GSK-J4 vs. LPS group, *P* < 0.01, LPS+*Adora2a* knockdown vs. LPS group, *P* < 0.01, LPS+*Adora2a* knockdown+GSK-J4 vs. LPS group). **B** Flow cytometry analysis for the evaluation of apoptosis after *Adora2a* knockdown in LPS-stimulated RAW264.7 cells: Compared to cells that were only exposed to LPS, cells that were further treated with GSK-J4, underwent knockdown of *Adora2a*, or were both treated with GSK-J4 and underwent *Adora2a* knockdown showed higher levels of apoptosis (*n* = 3 in each group, *P* < 0.01, LPS+GSK-J4 vs. LPS group, *P* < 0.01, LPS+*Adora2a* knockdown vs. LPS group, *P* < 0.01, LPS+*Adora2a* knockdown+GSK-J4 vs. LPS group). ns indicated no statistical significance, * *P* < 0.05, ** *P* < 0.01, and *** *P* < 0.001.

In vitro, LPS pre-treatment of RAW264.7 Cells transfected with an *Adora2a* knockdown construct was used to analyse the effect of JMJD3 and ADORA2A on macrophage apoptosis using western blots and flow cytometry. Western blot revealed that inhibiting JMJD3 with GSK-J4, knockdown of *Adora2a*, or both inhibiting JMJD3 and knockdown of *Adora2a* all increased cleaved-capsase3 expression compared to LPS-stimulated macrophages (Fig. 7A). Flow cytometry revealed that inhibiting JMJD3 with GSK-J4, knockdown of *Adora2a* in LPS-stimulated macrophages, or both inhibiting JMJD3 and knockdown of *Adora2a* all increased macrophage apoptosis compared to LPS-stimulated macrophages (Fig. 7B).

## Discussion

Gram-negative bacterial infection in the lung or intestines is known to be an important factor in the development of ALI, owing to the bacterially secreted endotoxins (mainly LPS) [17]. During ALI, there is a prominent increase in the number of macrophages due to disruption of the alveolar vascular epithelial barrier and inflammatory recruitment [18]. Promoting activated macrophage apoptosis to reduce the activated macrophage count can accelerate the resolution of inflammation and relief of ALI [3, 4]. Our results revealed that inhibiting JMJD3 can relieve the pathological performance of ALI mice as alveolar collapse and inflammatory cell infiltration accompany the remission of inflammation. JMJD3-induced H3K27me3 demethylation up-regulation of ADORA2A transcription exacerbates ALI by inhibiting apoptosis of macrophages. Furthermore, JMJD3 upmodulates the expression of ADORA2A through the JMJD3–H3K27me3-C/EBPβ-ADORA2A pathway. To the best of our knowledge, our study is the first to investigate the mechanisms by which JMJD3 regulates apoptosis in macrophages through ADORA2A in ALI.

Based on JMJD3’s involvement in the pro-inflammatory response of macrophages [19], we explore its mechanism as a histone demethylase in ALI mice and the possible therapeutic effects of its specific inhibitor GSK-J4. In mouse macrophages with ALI, we found that ALI remission after inhibition of JMJD3 was accompanied by a decrease in macrophage count, and we further explored the possible mechanism of JMJD3’s involvement in the apoptosis of macrophages. GSK-J4 as a specific inhibitor of JMJD3 has shown strong therapeutic potential in a number of diseases including liver fibrosis and tumours [20, 21]. Our study also suggests an important role for GSK-J4 in the treatment of ALI.

ADORA2A may involve in macrophage apoptosis upon LPS stimulation [13, 14, 22]. Our study showed that ADORA2A inhibits macrophage apoptosis in ALI mice. However, there are some incongruities between the previous study and ours regarding ADORA2A’s role in ALI. Aggarwal et al. reported that ADORA2A is protective against ALI after LPS and oxygen in *Adora2a*-knockout mice and in lung macrophages following exposure to LPS and oxygen [13]. We noted that different doses and types of LPS might cause the diversity results [23]. In our study, LPS was used with a higher dose. We investigated the ADORA2A *a*’s effect on ALI in 24 h, however, Aggarwal et al. investigated ADORA2A’s effect on ALI in 48 and 72 h [13]. During different time points of ALI, macrophages can function in either early induction or late resolution. [5, 24]. Besides, our study and Aggarwal et al. study both found a TNF-α level decrease in BAL at 24 h. Therefore, the conclusions were not contradictory and the effect of ADORA2A in macrophages should be further investigated.

To explore the possible regulation relationship between H3K27me3 and ADORA2A, we performed chromatin immunoprecipitation between H3K27me3 and *C/ebpβ* as well as between C/EBPβ and *Adora2a*. The results revealed that LPS-induced H3K27me3 demethylation increased the expression of *Adora2a*. C/EBPβ, as a transcription factor, is a regulator of multi-inflammatory factors in macrophages [25]. Moreover, C/EBPβ may be associated with macrophage apoptosis via the mammalian target of the rapamycin pathway [16]. C/EBPβ play an important role in ADORA2A expression in macrophage [26, 27], we found C/EBPβ was a transcription factor for *Adora2a, C/ebpβ* and *Adora2a* were both regulated by JMJD3-induced H3K27me3 demethylation in LPS-induced ALI.

Overall, upregulating the level of apoptosis of macrophages is important for ALI. GSK J4, a selective inhibitor of JMJD3, enhanced apoptosis and ameliorated inflammation in ALI via inhibition of H3K27 demethylation. In this study, we show that JMJD3 increased the expression of ADORA2A in the macrophage of ALI mice. ADORA2A is a negative regulator of cleaved caspase-3 in macrophages and GSK-J4 could inhibit ADORA2A in macrophages and successfully promote proinflammatory macrophage apoptosis. Therefore, there is a novel pathway for macrophage-mediated apoptosis and inflammation, which includes JMJD3-mediated demethylation of H3K27me3, upregulation of *C/ebpβ* and *Adora2a*, and suppression of caspase-3 cleavage. Interventions in this pathway such as GSK-J4 might be beneficial for the treatment of ALI.

There are some limitations that need to be acknowledged. Firstly, using intratracheal LPS to develop an ALI model could mimic ALI in humans more closely than other methods [28], especially when investigating monocytes–macrophages in the lungs [23]. Besides, the macrophage apoptosis and inflammatory reaction were also similar under LPS stimulation macrophages between mice and humans including *C/ebpβ* and *Adora2a*, the main proteins in our study [29, 30], it was not completely consistent between them [31]. Human sample experiments should be performed further. Referring to previous studies [8, 32], we chose a pre-treatment for ALI mice using GSK-J4, but this method may not adhere to the clinical treatment of ALI patients.

In conclusion, JMJD3 might contribute to inhibited apoptosis of macrophages to aggravate the inflammatory reaction targeting ADORA2A in LPS-induced ALI. Inhibiting JMJD3 may alleviate inflammatory reaction in LPS-induced ALI targeting enhancement of macrophages’ apoptosis.

## Materials and methods

### Animals and treatments

Male mice were more likely to develop ALI [28] and female mice performed differently than male mice due to a better tolerance for sepsis [33]. Therefore, we chose male C57BL/6j mice (aged 8–12 weeks, weight 18–22 g) (HFK Bioscience Co. Ltd, Beijing, China) and raised the mice in a pathogen-free environment (Shengjing Hospital of the China Medical University, Centre laboratory) under a 12-h/12-h dark/light cycle and provided ad libitum access to food and water.

ALI model for mice was built by a method of intraperitoneal LPS (*Escherichia coli*, 0111: B4, Sigma, USA). LPS (*Escherichia coli*, 0111: B4, Sigma, USA) was dissolved in PBS to achieve a final concentration of 1 mg/ml. 2.5 mg/kg of this LPS solution was administered intratracheally to the mice to establish an ALI model. Besides, two kinds of inhibitor for JMJD3 (GSK-J4, ab144395, Abcam, Cambridge, UK) and SCH-58261 (an antagonist of *Adora2a*, ab120439, Abcam, Cambridge, UK) were dissolved with dimethyl sulfide (DMSO) and used for the treatment of ALI mice. A total of 40 mice were divided into four groups the control group, the LPS group, the GSK-J4 treatment group (LPS+GSK-J4 group) and the *Adora2a* antagonist+ LPS group, containing 10 mice each. LPS was dissolved in PBS to achieve a final concentration of 1 mg/ml. 2.5 mg/kg of this LPS solution was administered intratracheally to the mice to establish an ALI model in the LPS group, GSK-J4 treatment group and *Adora2a* antagonist+ LPS group after sedation with isoflurane. After intratracheal LPS, 10 mg/kg GSK-J4 was administered intraperitoneally at the same time and 5 mg/kg/day of GSK-J4 was injected until they were sacrificed. In the *Adora2a* antagonist group, 2 µg/kg/day SCH-58261 was administered intraperitoneally after intratracheal LPS. In the control group, the same volume of PBS was administered intraperitoneally and the same volume of DMSO was administered intratracheally.

After 24 h, peripheral blood was drawn for enzyme-linked immunosorbent assay (ELISA) and left lung tissues were used for hematoxylin and eosin (H&E) staining and immunofluorescence. Bronchoalveolar lavage (BAL) was performed with 0.8 mL PBS instillation three times using the right lung tissues.

### Histological staining and evaluation of lung injury

Mouse lung tissues were fixed in 4% paraformaldehyde and serial 4-μm-thick paraffin-embedded sections were used for H&E staining. The severity of ALI was evaluated using a score developed from specific histopathologic findings on light microscopy. Four histopathologic signs were used for this score including hyperaemia, haemorrhage, oedema of the alveolar wall, and inflammatory cell infiltration. Each sign was graded from 0 to 4 (0 = normal, 1 = very small amount, 2 = mild, 3 = moderate, 4 = severe, 5 = very severe) [17]. The total score was the summation of all item scores, and it was used to evaluate the degree of lung injury. Pathological changes were observed under a light microscope.

### Immunofluorescence assay

Mouse lung tissues were fixed in 4% paraformaldehyde and serial 3-μm-thick paraffin-embedded sections were used for immunofluorescence. The samples were deparaffinized and rehydrated, and lung sections were blocked using 1% sheep serum at room temperature for 1 h. Next, the sections were incubated with anti-JMJD3 (1:200, NBP1-06640, Novus, USA), anti-H3K27me3 (1:200, ab192985, Abcam, Cambridge, UK), anti-C/EBPβ (1:200, 23431-1-AP, Proteintech, USA), anti-cleaved caspase 3 (1:200, 9664S, CST, Boston, USA), anti-F4/80(ab16911, Abcam, Cambridge, UK), anti-ADORA2A (1:200, ab34611, Abcam, Cambridge, UK), and anti-H3K27me3 (ab6002, Abcam, Cambridge, UK) antibodies overnight at 4 °C. The next day, the sections were incubated with the appropriate secondary antibodies (1:200, ab15007, ab150079, ab150160, and ab150115; Abcam, Cambridge, UK) for 2.5 h at room temperature. Subsequently, the samples were incubated with 4′,6-diamidino-2-phenylindole (DAPI) to label the nuclei. Besides, the cells in lung tissue with F 4/80+ were recognized as macrophages. Immunofluorescence was observed under a fluorescence microscope (Olympus, Tokyo, Japan). Different groups of lung tissue were observed in the same settings and differences among groups were compared. Image-Pro Plus was used for image merging and was semi-quantitatively analysed by the ImageJ software (NIH, Bethesda, MD, USA).

### TUNEL assay

Lung tissue sections were immunoassayed with F4/80 to identify macrophages. Terminal deoxynucleotidyl transferase-mediated dUDP nick-end labelling (TUNEL) assay was used to analyse the macrophage apoptosis. Lung tissue slices permeabilized with protein kinase K at room temperature for 10 min were used for the TUNEL assay (abs5003; Absin, Shanghai, China). Next, permeabilized slices were incubated in DNase I buffer at room temperature for 5 min. After aspirating the buffer, slices were incubated with DNase I (20 U/mL in DNase I buffer) at room temperature for 10 min. The slices were then incubated with TdT enzyme (1 μL/50 μL TUNEL equilibrium buffer) at room temperature for 10 min. Finally, DAPI staining was performed to label the nuclei. The apoptotic cells were calculated by the number of positively staining cells of cells per microscopic field and were observed under a fluorescence microscope in F4/80+ cells (Olympus, Tokyo, Japan).

### Cell growth and treatment

Macrophages (RAW 264.7) were purchased from the Chinese Academy of Science (Shanghai, China), cultured in Dulbecco’s minimal essential medium (DMEM) (10% FBS, Gibco, USA), and maintained at 37 °C in 5% CO_2_.

Cells cultured with 100 ng/ml LPS in DMEM for 24 h were designated as the LPS group. Cells pre-treated with GSK-J4 (10 μM for 24 h) and then cultured sequentially with 100 ng/mL LPS and 10 μM GSK-J4 for 24 h were labelled as the LPS group+GSK-J4 group. Cells labelled as the control group was added to the same volume of dimethyl sulfoxide (GSK-J4 dissolvent) and PBS (LPS dissolvent).

### ELISA

Enzyme-linked immunosorbent assay (ELISA) was performed in 96-well plates. IL1β, IL6, and TNF-α levels were detected in the serum and BAL fluid collected from mice and in the supernatant of cell culture media (CUSABIO; CSB-E08054m for IL1β, CSB-E0439m for IL6, and CSB-E04741m for TNF-α, Wuhan, China).

### Western blot analysis

RIPA lysis buffer, histone extraction kit (ab113476; Abcam, Cambridge, UK), and ExKine™ Nuclear and Cytoplasmic Protein Extraction Kit (KTP3001, Abbkine, Wuhan, China). Anti-JMJD3(1:1000, NBP1-06640, Novus, USA), anti-H3K27me3(1:1000, ab192985, Abcam, Cambridge, UK), C/EBPβ (1:1000, 23431-1-AP, Proteintech, USA), anti-cleaved-Caspase3 (1:1000, 9664S, CST, Boston, USA) and anti-ADORA2A (1:1000, ab34611, Abcam, Cambridge, UK) primary antibodies were used. *β*-actin (1:5000, AF7018, Affinity, USA) and H3 (1:1000, AF0863, Affinity) were used as controls. Protein samples were separated by sodium dodecyl sulfate-polyacrylamide gel electrophoresis and transferred to polyvinylidene difluoride (PVDF) membranes. Non-specific binding on PVDF membranes was blocked with 5% non-fat powdered milk in Tris-buffered saline and Tween-20 at room temperature for 2 h. The membranes were incubated overnight with primary antibodies at 4 °C and with secondary antibodies the following day for 2 h at room temperature. Finally, the ECL kit was used for chemiluminescence detection. The grey scale of the target band was detected in a Western blot developing device. The differences among different groups were compared based on triple repetition, with an *β*-actin internal reference and analysed using the ImageJ software (NIH, Bethesda, MD, USA).

### Quantitative polymerase chain reaction

For quantitative real-time quantitative polymerase chain reaction (qRT-PCR) analysis, RNA was isolated from RAW264.7 cells using Trizol and reverse-transcribed with Superscript II using an oligo(dT) primer. The resulting cDNA (5 times diluted) served as a template for qRT-PCR, which was performed on a Roche 480 PCR system with SYBR Green. β-Actin was used as the endogenous control, and relative gene expression was calculated using the 2^−ΔΔct^ method. The primers used for the amplification of the target genes (e.g.*, Jmjd3, β-actin*, *Adora2a*, *and C/ebp β*) are listed in Table 1.

**Table 1:** PCR primer sequences.

Gene	Forward (5′–3′)	Reverse (5′–3′)	Length
*β-actin*	CTGTCGAGTCGCGTCCACCC	ACATGCCGGAGCCGTTGTCG	128 bp
*C/ebpβ*	GACAAGCTGAGCGACGAGTA	GCTTGAACAAGTTCCGCAGG	196 bp
*Adora2a*	GCCATCCCATTCGCCATCA	GCAATAGCCAAGAGGCTGAAGA	122 bp
*Jmjd3*	CCACTGCTGGAGTGCTTGTTC	GAAAGCCAATCATCACCCTTGTC	91 bp

*PCR* polymerase chain reaction.

### Cell immunofluorescence assay

RAW264.7 cells were cultured in 24-well plates for immunofluorescence. The cells in each well were fixed in 4% paraformaldehyde and permeabilized with 0.1% Triton-100 at room temperature for 15 min. The subsequent methods were performed as described above for the lung tissue immunofluorescence assay.

### Chromatin immunoprecipitation-PCR

Chromatin immunoprecipitation (ChIP) was performed using the ChIP-IT® Express Enzymatic kit (Active Motif, USA). RAW264.7 cells were crosslinked with 4% formaldehyde and de-crosslinked with glycine. The cells were collected, digested, and lysed. An enzymatic shearing cocktail (diluted with ddH_2_O and glycerol) was used to fragment the chromatin. Previous experiments determined the appropriate shearing time to cut chromatin into 100–500 bp fragments to be 5 min and 30 s. EDTA was added to terminate the enzymatic shearing. Magnetic beads were coupled to ChIP-antibodies (anti-H3K27me3/anti-C/EBPβ/IgG (31887, Thermo Fisher Scientific, Waltham, MA, USA)) via rotation at 4 °C overnight. The following day, the magnetic beads were eluted and de-crosslinked to capture the protein-DNA complexes. Before mixing the magnetic beads and ChIP-antibodies, the primary protein-DNA complexes were extracted and diluted in ChIP-buffer, proteinase K, and proteinase K stop buffer, to obtain the ‘input’ protein-DNA complexes. Two groups (control and LPS) were partitioned for each ChIP-PCR assay. The corresponding input and IgG fractions were used to perform the PCR reactions. The input fraction was used as an endogenous control and the relative gene expression for each sample was calculated using the 2^−ΔΔct^ method. Two ChIP-antibodies, anti-H3K27me3 and anti-C/EBPβ, were used to extract the protein-DNA complexes for qPCR with the objective primers *Adora2a* and *C/ebp β*. The primers used for the amplification of the target genes are listed in Table 2.

**Table 2:** ChIP-PCR primer sequences.

Gene	Forward (5′–3′)	Reverse (5′–3′)	Length
*C/ebpβ*	ATCAGGGGGATCAAACGAGT	TCTGCTGTGTGACCTTAGCC	122 bp
*Adora2a*	TCGCCATCCGAATTCCACTC	TTTGTGCCCACAGATCTAGCC	71 bp

*ChIP-PCR* chromatin immunoprecipitation-polymerase chain reaction.

### Cell transfection

RAW264.7 Cells (5 × 10^4^) were seeded into 24-well plates and cultured for 24 h in complete culture media supplemented with 100 ng/mL LPS. Next, the cells were transfected with an *Adroa2a*-knockdown-lentivirus at a multiplicity of infection MOI of 100 for 24 h (Hanbio, Shanghai, China). After transfection, infected cells were cultured for 48 h for western blotting and other experiments.

### Flow cytometry

To detect apoptosis, RAW264.7 cells (1 × 10^5^) were seeded into 6-well plates and subjected to flow cytometry using the Annexin V-FITC/PI apoptosis detection kit (BD Biosciences, San Diego, CA, USA) according to the manufacturer’s recommendations. Cells from different groups (3 × 10^5^ cells/well) were seeded and re-suspended in the binding buffer. After staining with annexin V-FITC and PI for 15 min in the dark, the cells were analysed by flow cytometry (BD Biosciences) using FlowJo analysis software (FlowJo, LLC, Ashland, Ore).

### Statistical analysis

Continuous variables were expressed as mean ± SD and tested for significance using one-way analysis of variance ANOVA with Tukey’s multiple comparisons post-test. Statistical analyses were performed using GraphPad Prism 6 (GraphPad Software, La Jolla, CA, USA). Results with *P*-value < 0.05 were considered statistically significant (* indicates *P* < 0.05, ** indicates *P* < 0.01, *** indicates *P* < 0.001).

## Supplementary information

Full and uncropped western blots

## Data Availability

The datasets used or analysed during the current study are available from the corresponding author on reasonable request.
