# Supplementary figures and images for: H3K27 tri-demethylase JMJD3 inhibits macrophage apoptosis by promoting ADORA2A in lipopolysaccharide-induced acute lung injury
Source: Cell Death Discov. 2022 Dec 1;8:475. doi: 10.1038/s41420-022-01268-y (PMC9715944; doi:10.1038/s41420-022-01268-y)

**JMJD3**

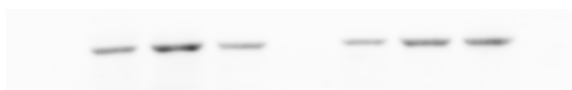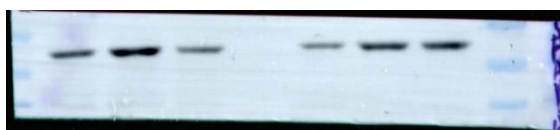

**$\beta$ -actin**

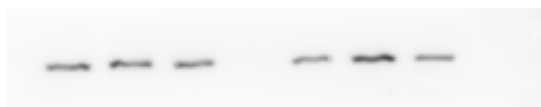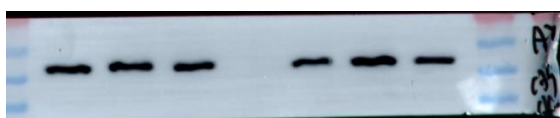

**H3K27me3**

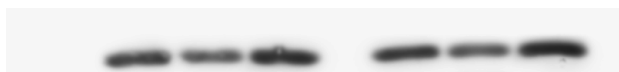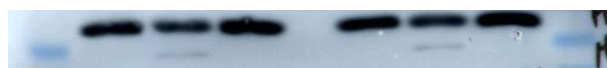

**H3**

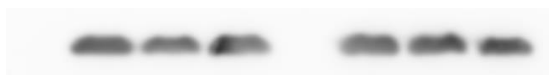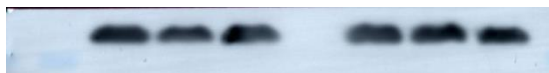

**C/EBP $\beta$**

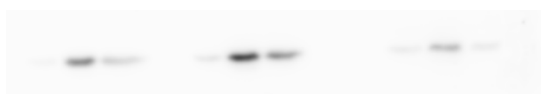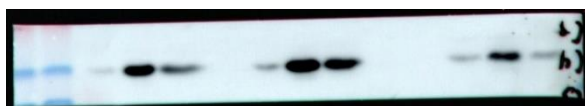

**$\beta$ -actin**

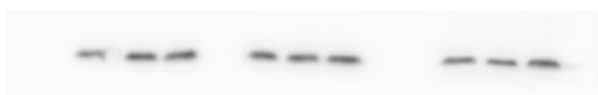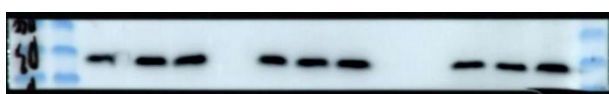

## ADORA2A

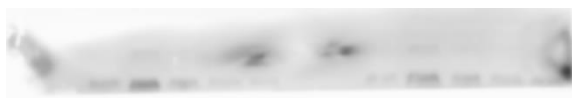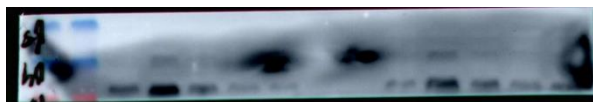

## Cleaved caspase 3

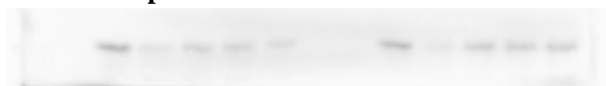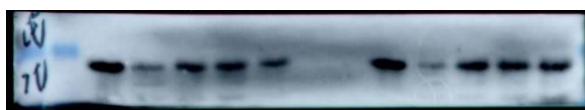

## $\beta$ -actin

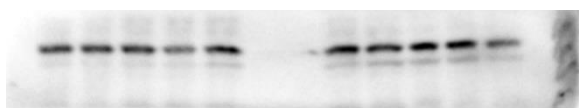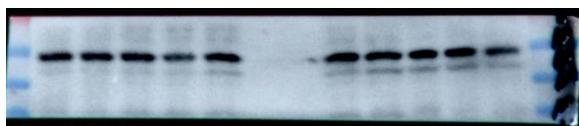

Supplement: Supplementary file 1 — Full and uncropped western blots [file 41420_2022_1268_MOESM1_ESM.pdf]
